# Theoretical Study of the Structure and Binding Energies of Dimers of Zn(II)-Porphyrin Derivatives

**DOI:** 10.1021/acs.jpca.2c03692

**Published:** 2022-10-04

**Authors:** Sule Atahan-Evrenk

**Affiliations:** Faculty of Medicine, TOBB University of Economics and Technology, Sogutozu Cd No. 43 Sogutozu, Ankara06560, Turkey

## Abstract

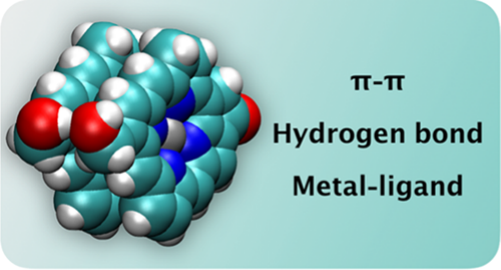

Zinc-complexed porphyrin and chlorophyll derivatives form functional aggregates with remarkable photophysical and optoelectronic properties. Understanding the type and strength of intermolecular interactions between these molecules is essential for designing new materials with desired morphology and functionality. The dimer interactions of a molecular set composed of porphyrin derivatives obtained by substitutional changes starting from free-base porphyrin is studied. It is found that the B97M-rV/def2-TZVP level of theory provides a good compromise between the accuracy and cost to get the dimer geometries and interaction energies (IEs). The neglect of the relaxation energy due to the change in the monomer configurations upon complex formation causes a more significant error than the basis set superposition error. The metal complexation increases the binding energy by about −6 to −8 kcal/mol, and the introduction of keto and hydroxy groups further stabilizes the dimers by about −20 kcal/mol. Although the saturation of one of the pyrrol double bonds does not change the IE, the addition of R groups increases it.

## Introduction

Metalloporphyrins are ubiquitous in light-harvesting organisms.^1^ Because of their extraordinary photophysical and optoelectronic properties, synthetic analogues that mimic these systems draw interest with potential applications in solar cells, sensors, or photodynamic therapy.^2−5^ One of the systems that inspired a myriad of work is the self-assembled supramolecular structures of bacteriochlorophylls (BChls), which form highly efficient antennae to capture light without a protein scaffold to hold the structures together.^6−8^ Replacing the central magnesium atom in BChl *c* with zinc has been a rewarding synthetic strategy to increase the stability of the metal complexes.^3^ Moreover, by chemical modifications of the structures, their self-assembly processes and morphologies can be controlled.^9^

Like their BChl counterparts, zinc-chlorins have rich intermolecular interaction types: π–π stacking, H-bonding, and metal–ligand.^10^ An accurate description of intermolecular interactions is crucial for understanding the forces under which the supramolecular aggregates form.^11^ Such descriptions also provide the necessary benchmark for improving the available force fields.^12^ However, the studies on the intermolecular interactions governing the dimer formation of these zinc-porphyrins are scarce. Previously, Kuznetsov^13^ analyzed ground-state geometries of Zn-porphyrin dimers and found good agreement with the experimental crystal structure. To the best of our knowledge, the relative importance of different types of interactions and the effect of the substitutions on the interaction energy (IE) strength are not known.

Here, for the first time, we present a systematic study of the IE in zinc-porphyrin complexes with increasing structural complexity so that the effect of substitutions on the dimer IEs is quantified. We used semiempirical, density functional, and symmetry-adapted perturbation methods to find a good balance of cost and accuracy. Moreover, the types of intermolecular interactions are evaluated using energy decomposition analysis based on the symmetry-adapted perturbation theory (SAPT).

**Figure 1:**
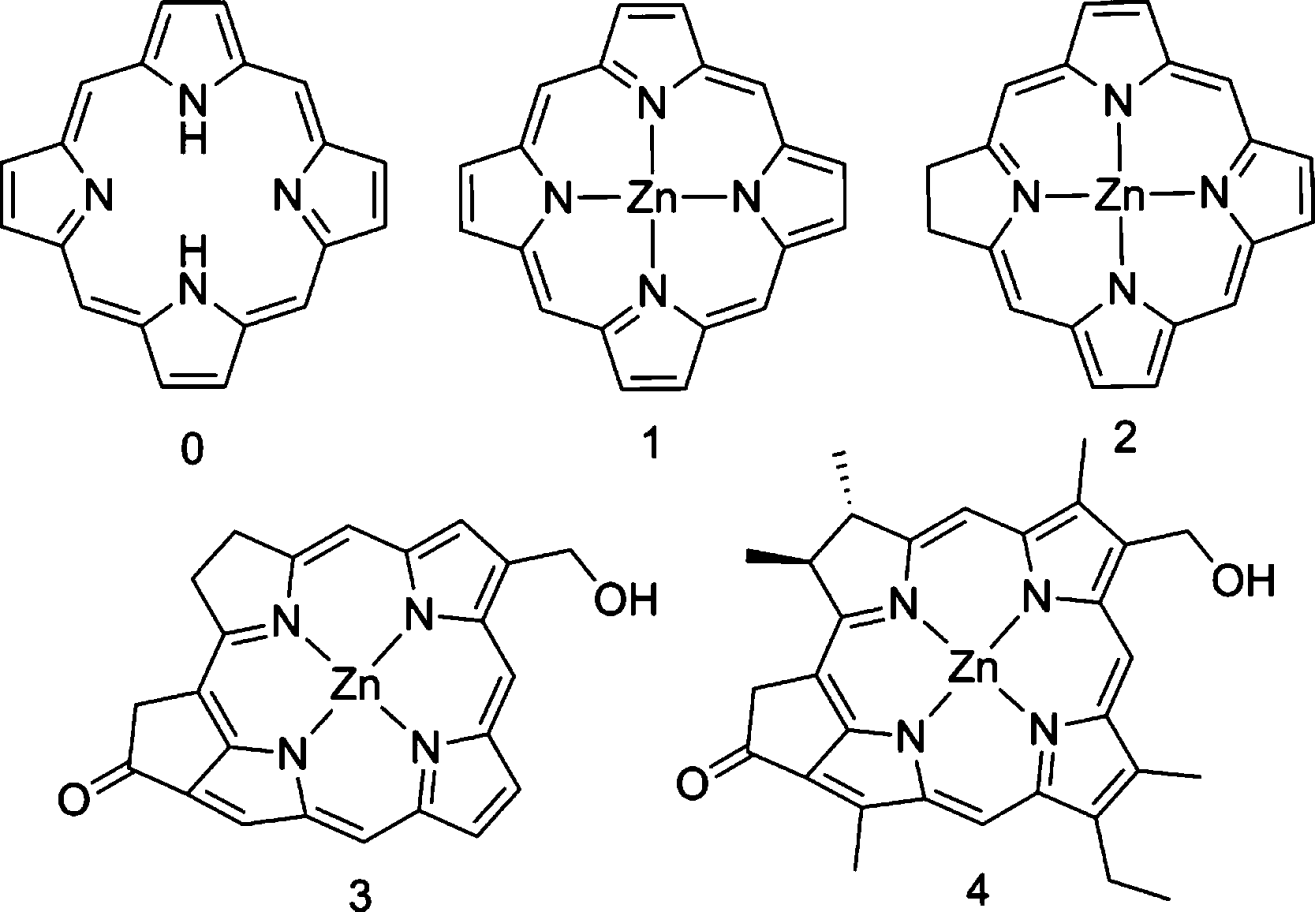
Molecules studied in this work.

A set of compounds starting from the simplest free-base porphyrin to more complex substituted Zn-chlorins are studied (Figure 1). Although the focus is on the metal complexed structures, the free-base porphyrin (**0**) is included as a reference. Molecule **1** is a highly symmetrical Zn(II)-porphyrin for which an experimental crystal structure is available.^14^ In molecule **2** (Zn-chlorin), the 17–18 bond is saturated, and hence, there are 20 π electrons. Molecule **3**, similar to BChl *c*, has a hydroxy group attached to the 3^1^ position, and the 13th, 14th, and 15th carbons are a part of a cyclopentenone. Molecule **4** has all of the methyl and ethyl group substitutions seen in BChl. This setup makes it possible to distinguish various groups’ chemical and sterical contributions to the dimer IEs.

## Methods

For van der Waals complexes, the geometry relaxation upon complexation is small, changes in the interatomic distances are usually less than 10^–2^ Å. Hence, the dimer IEs can be calculated with frozen monomer geometries. However, the amount of geometrical relaxation changes from one method to the other among the methods used here. For example, for some of the semiempirical methods, monomer interatomic distances change as much as 10^–1^ Å upon dimer formation. In addition, unlike van der Waals complexes, the dimers studied have metal–ligand interactions with distances <3 Å. Therefore, both the binding energy (BE) and IE are calculated.

The BE, which is the total dimer energy with respect to the optimized monomer asymptote is
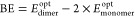


The IEs for the dimers are calculated as follows
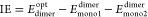


Here, the monomer energies for their geometry in the dimer are subtracted from the total dimer energy. The basis set superposition error (BSSE) correction is obtained by the counterpoise method of Boys and Bernardi.^15^

The performance of the density functional theory (DFT) methods is investigated for a number of functionals. The long-range corrected functional ωB97M-V^16^ performed better than many others for a large number of data points.^16^ Moreover, together with the def2-TZVP basis set,^17^ it described the chemistry of transition metal complexes successfully.^18^ For molecule **1**, the results from the ωB97M-V functional is compared with the results from other density functionals from different rungs. These are B97-D3^19^ from rung 1, TPSS-D3(BJ)^20,21^ and B97M-rV^22^ from rung 2, M06-2X^23^ with and without -D3 dispersion, MN15,^24^ ωB97X-D,^25^ ωB97X-D3,^26^ and ωB97X-V^27^ from rung 3.

In addition, as low-cost alternatives to the methods mentioned above, the three corrections composite methods B97-3c^28^ and PBEh-3c^29^ and semiempirical methods DFTB3-D4^30,31^ and GFN2-xTB^32^ are investigated. B97-3c is a low-cost variant of B97-D that provides dispersion and avoids basis set errors with an optimized triple zeta basis (mTZVP). PBEh-3c yields geometries that are close to MP2/TZ accuracies with a fraction of the computational expense. DFTB3-D4 is a tight-binding approach that is also suitable for solid-state structure calculations. GFN2-xTB is also a semiempirical tight-binding method with multipole electrostatics and dispersion, which shows promise in the optimization of large aggregates and complexes of transition metals.

An essential technique for analyzing the noncovalent interactions is the SAPT.^33^ The SAPT IE is a collection of the electrostatic, exchange, induction, and dispersion components. Within the SAPT formulation, under the assumption that intermolecular forces are much weaker than the intramolecular covalent interactions, the distortions in the geometry of a molecule in the presence of another one could be assumed negligible. Then, the IE is calculated as a perturbation in the monomer basis. If all of the higher-order terms in the perturbation expansion are included, it is possible to achieve accuracy close to that of CCSD(T). However, the standard level expansion (SAPT2) scales similarly to CCSD(T), ≈*N*^7^. So instead, here, the SAPT(DFT)^34^ method together with the density-fitting approximation with frozen core orbitals^35^ is used (the grac shift: 0.2498). Unfortunately, the def2-TZVP basis could be used only for molecules **0–2** due to the large basis set size and memory limitations (5478 basis functions for molecule **3**).

The DFT calculations are performed as implemented in Q-Chem 5.3^36^ with BrianQC 1.1^37^ as the GPU interface. The DFTB3 method with 3ob-3-1 Slater–Koster parameters^38,39^ (Hubbard derivatives: Zn = −0.03, O = −0.1575, N = −0.1535, C = −0.1492, and H = −0.1857) and with D4 dispersion (a1 = 0.5523240, a2 = 4.3537076, s6 = 1, s8 = 0.6635015, and s9 = 1) is used as implemented in the DFTB+^40^ package. For GFN2-xTB, the xTB code,^41^ for B97-3c and PBEh-3c methods, ORCA,^42^ and for SAPT(DFT), Psi4^43^ programs are used. The molecular visualizations are performed using VMD.^44^

## Results and Discussion

To benchmark the accuracy of the computational methods, we first focus on the available Zn(II) porphyrin crystal structures.^14^ Two crystal structures with *P*1̅ and *P*2_1_/*c* symmetries are available, both of which feature parallel displaced molecular packing in the form of dimer and trimers due to strong π–π stacking interactions. The monomers are relatively close-packed, with the distances between the planes being about 3.2 Å, which are shorter than what was reported for dimers of free-base porphyrin (3.4 Å),^45^ and this perhaps is a result of the interaction of the metal atom with the nitrogen of the pyrrole ring in the neighboring monomer. This coordinative interaction pulls the metal off-center and closer to the other monomer, doming the N–Zn–N angles to 172–174°. The highly symmetric gas-phase structure of Zn-porphyrin (*D*_4*h*_) is no longer in the crystal due to intermolecular interactions. For example, there is an asymmetry in the intramolecular Zn–N bond distances.

First, we optimized the dimer structure extracted from the *P*2_1_/*c* crystal^14^ by the ωB97M-V^16^ method using the def2 basis family.^17^ To make sure that the BSSE does not lead to an artificially high IE, we performed the dimer geometry optimization with def2-SVP, -TZVP, and -QZVP bases.^17^ The difference in the IEs for triple and quadrupole zeta bases is small (Table 1). This finding confirms the results of earlier studies, which showed that the def2-TZVP basis is reliable without the counterpoise correction.^18,46^

**Table 1:** BE and Some of the Geometrical Features for the Monomer and Dimer Optimized at the ωB97M-V Level of Theory with Karlsruhe Basis Sets (kcal/mol) [m: Monomer; d: Dimer; Prime Indicates the Atoms of the other Monomer in the Dimer; “-” Indicates the Minimum and Maximum Distances; and All Distances Are in Angstrom (Å)]

method	BE	Zn–N (m)	Zn–N (d)	Zn–N′	Zn–Zn′	N–Zn–N (deg)
def2-SVP	–41.6	2.05	2.05–2.10	2.58	3.10	165
def2-TZVP	–30.2	2.04	2.04–2.06	2.84	3.35	170
def2-QZVP	–29.2	2.04	2.04–2.06	2.89	3.38	171
experimental	-		2.03–2.05	2.97	3.54	171

The BSSE in the IE for the dimer optimized at the ωB97M-V/def2-TZVP level of theory is also calculated (Table 2). As expected, the larger the basis set, the smaller the error. The BSSE with def2-TZVP is approximately 2 kcal/mol. Should the BSSE be assumed to be similar for both the BE and IE calculations, the difference between the IE and BE is the geometry relaxation energy due to the geometrical deformation of the monomers in the dimer. Hence, the relaxation energy is approximately 3 kcal/mol compared to 2 kcal/mol BSSE. Therefore, ignoring the monomer geometry relaxation upon complex formation may not be justifiable if high accuracy is necessary.

**Table 2:** IE, BSSE-Corrected IE (IE_c_), and BSSE Corrections for the Dimer Geometry Optimized at the ωB97M-V/def2-TZVP Level of Theory (kcal/mol)

basis	IE	IE_c_	BSSE
def2-SVP	–43.3	–30.7	12.6
def2-TZVP	–33.2	–31.3	1.9
def2-QZVP	–32.1	–31.6	0.5

Overall, the computed geometries show a good agreement with the crystal structures (Table 1). Note that only the shortest Zn–N′ distances and the smallest N–Zn–N angles are reported in the tables. Similar to the crystal structure, the monomer is no longer symmetrical; there is a range of bond lengths and angles for the molecules in the dimer. For example, the intramolecular Zn–N distances vary in the range of 2.03–2.05 Å. In addition, there is a sufficient overlap of the optimized geometry for the dimer with the initial geometry extracted from the crystal. The high computational cost of using a quadruple-zeta basis is not justified since it does not significantly improve the geometry or the IE.

**Figure 2:**
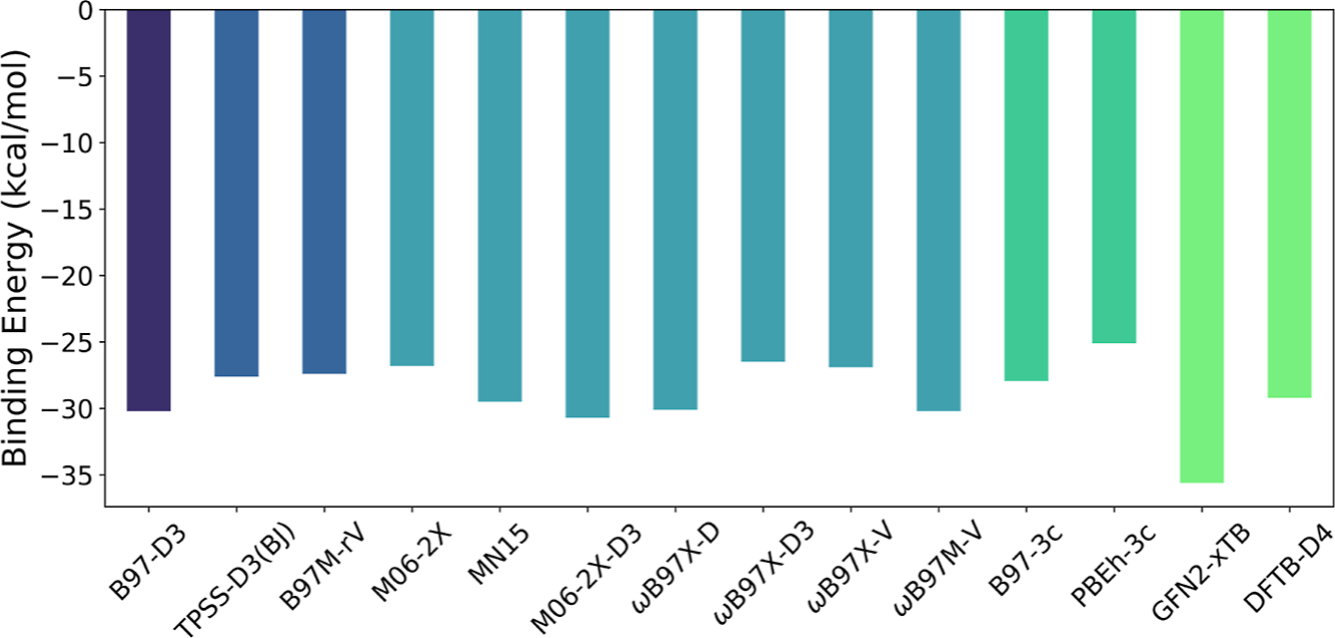
BEs for the dimer (**1**) obtained using various methods; all DFT calculations are performed with the def2-TZVP basis set.

The geometry and BE of the dimer (molecule **1**) obtained using ωB97M-V/def2-TZVP is compared with those obtained using other methods in Figure 2 and Table 3. All of the DFT results are approximately within 3 kcal/mol of each other. Previously, Kuznetsov studied the Zn-porphyrin dimer geometry using ωB97XD (Grimme’s D2 dispersion model) with the def2-TZVP basis set and reported a BE in the same range (−27.3 kcal/mol) as that for molecule **1**.^13^

**Table 3:** BEs for the Dimer (**1**) Obtained Using Various Methods in kcal/mol [m: Monomer, d: Dimer, and Prime Indicates the Atoms of the other Monomer in the Dimer; All Distances Are in Angstrom (Å); and DFT Calculations Are Performed with the def2-TZVP Basis Set]

method	BE	Zn–N (m)	Zn–N (d)	Zn–N′	Zn–Zn′	N–Zn–N (deg)
B97-D3	–30.2	2.06	2.06–2.08	2.96	3.45	172
TPSS-D3(BJ)	–27.6	2.05	2.05–2.08	2.70	3.21	168
B97M-rV	–27.4	2.04	2.03–2.05	2.92	3.39	171
M06-2X	–26.8	2.05	2.03–2.08	2.78	3.29	169
MN15	–29.5	2.04	2.04–2.07	2.74	3.22	168
M06-2X-D3	–30.7	2.05	2.03–2.08	2.78	3.29	169
ωB97X-D	–30.1	2.04	2.04–2.05	3.06	3.52	174
ωB97X-D3	–26.5	2.04	2.04–2.05	3.16	3.63	175
ωB97X-V	–26.9	2.04	2.04–2.06	3.01	3.50	173
ωB97M-V	–30.2	2.04	2.04–2.06	2.84	3.35	170
B97-3c	–27.9	2.05	2.05–2.07	2.85	3.36	170
PBEh-3c	–25.1	2.04	2.04–2.07	2.76	3.28	169
GFN2-xTB	–35.6	2.03	2.03–2.07	2.42	2.89	163
DFTB-D4	–28.9	2.08	2.07–2.18	2.53	3.13	165
experimental			2.03–2.05	2.97	3.54	171

The approximate methods provide good accuracy while reducing the computational cost significantly (Table 3). The “-3c” methods provide a good geometry, whereas the GFN2-xTB optimization brings the monomers too close together, reflected as the over-doming in the N–Zn–N angle, resulting in a larger IE. One notable feature in DFTB-D4 is the intramolecular Zn–N distances being too far. Overall, the parameterized methods provide good accuracy for geometry optimization. Most of the IEs lie between −25 and −30 kcal/mol, the GFN2-xTB being the most significant deviation with a value of −35 kcal/mol. As a result of this comparison, to study the rest of the molecules, the following methods are chosen: B97M-rV, B97-D3, B97-3c, GFN2-xTB, and DFTB-D4.

**Figure 3:**
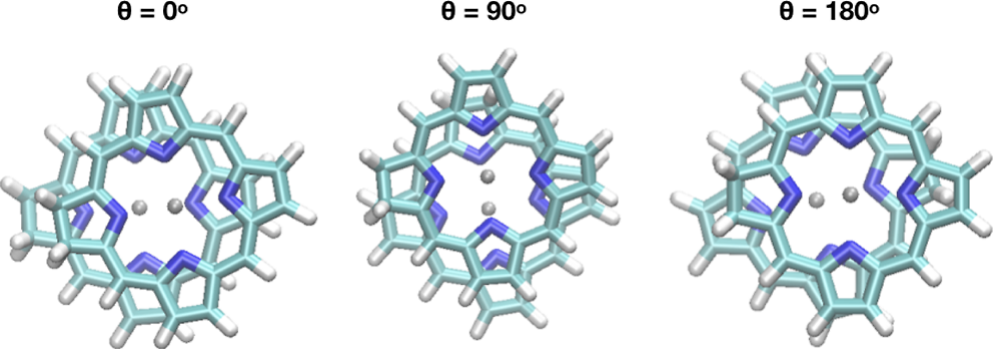
Geometries optimized at the level of B97M-rV/def2-TZVP for the dimers for molecule **2**.

For molecule **2**, three parallel-stacked initial structures, which were defined according to the alignment of the saturated bond (i.e., θ = 0° when the saturated bond coincides in the two dimers) were used. The optimized geometries are close to these initial structures, but with parallel displacement similar to that in molecule **1** (Figure 3).

The optimized geometries at various levels of theory have similar BEs (Table 4). DFTB-D4 and GFN2-xTB predictions for the intermolecular Zn–Zn′ and N–Zn′ distances are slightly shorter, and the N–Zn–N angles have more doming. The GFN2-xTB BE is about 5 kcal/mol larger than the B97M-rV value, similar to molecule **1**. Overall, the parameterized methods compare remarkably well with the B97M-rV/def2-TZVP level of calculations with a much lower cost. The similarity of the BE for rotated configurations of **2** indicates that a “sombrero hat” type of a potential energy surface is at play, as suggested previously by Mück-Lichtenfeld and Grimme for free-base porphyrin.^46^

**Table 4:** BEs (kcal/mol) for Dimers of Molecule **2** for Three Different Orientations [m: Monomer, d: Dimer, and Prime Indicates the Atoms of the Other Monomer in the Dimer; All Distances Are in Angstrom (Å); and DFT Calculations Are Performed with the def2-TZVP Basis Set]

method	BE	Zn–N (m)	Zn–N (d)	Zn–N′	Zn–Zn′	N–Zn–N (deg)
B97-D3						
0°	–29.2	2.03–2.15	2.03–2.18	2.96	3.50	171
90°	–31.1	2.03–2.15	2.04–2.14	3.12	3.50	175
180°	–30.7	2.03–2.15	2.04–2.16	3.19	3.58	175
B97M-rV						
0°	–25.1	2.01–2.12	2.01–2.15	2.90	3.41	171
90°	–26.9	2.01–2.12	2.02–2.10	3.07	3.42	174
180°	–26.6	2.01–2.12	2.01–2.13	3.15	3.50	174
B97-3c						
0°	–27.7	2.02–2.13	2.03–2.13	2.81	3.34	169
90°	–28.5	2.02–2.13	2.03–2.12	3.05	3.44	173
180°	–27.4	2.02–2.13	2.02–2.14	3.14	3.57	175
DFTB-D4						
0°	–27.8	2.05–2.19	2.05–2.29	2.50	3.16	164
90°	–27.8	2.05–2.19	2.05–2.28	2.57	3.17	166
180°	–26.6	2.05–2.19	2.04–2.28	2.61	3.22	166
GFN2-xTB						
0°	–32.8	2.01–2.11	2.02–2.16	2.42	2.92	162
90°	–33.4	2.01–2.11	2.02–2.11	2.57	2.90	165
180°	–31.1	2.01–2.11	2.02–2.14	2.63	2.93	166

**Figure 4:**
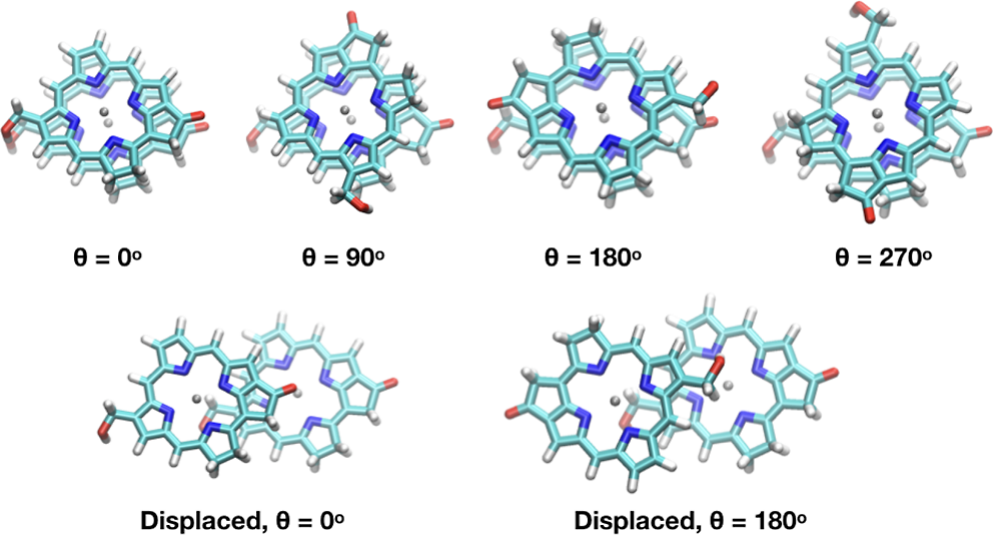
Initial geometries for the dimers for molecule **3**.

Molecule **3** includes both a hydroxy group and a ketone group; hence, in addition to π–π stacking, H-bonding and Zn–O interactions play a role in dimer formation. A total of six dimer geometries were used as the initial structures (Figure 4). These were the four parallel-stacked dimers, rotated θ = 0, 90, 180, and 270° degrees around the Zn–Zn′ axis, and two parallel-displaced geometries (initially displaced by about 6 Å) so that either hydroxy or keto oxygen on one monomer can interact with the metal atom on the other monomer. The 180° dimer with the double hydrogen bond has the strongest binding across the methods.

Again, optimized geometries of these dimers formed by rotation are all slightly sideways displaced, similar to molecules **1** and **2**. All of the final geometries are closer to the initial structure, indicating that the basin for the minima on the potential energy surface is wide. Consistently across the methods, the higher BE is for the dimer in which the θ = 180° rotation allows for two hydrogen bonds. As expected from H-bonding, the IE of these dimers is higher by about 3–4 kcal/mol per bond. The θ = 0° configuration has a weaker hydrogen bond, as reflected in the O–O distances in Table 5.

**Table 5:** BEs (kcal/mol) and Geometric Parameters for Dimers of Molecule **3** for Different Initial Orientations [m: Monomer, d: Dimer, and Prime Indicates the Atoms of the Other Monomer in the Dimer; All Distances Are in Angstrom (Å); and DFT Calculations Are Performed with the def2-TZVP Basis Set]

method	BE	Zn–N (m)	Zn–N (d)	Zn–Zn′	Zn–N′	N–Zn–N (deg)	H-bond	OH–O	Zn–O
B97-D3									
0°	–39.0	1.99–2.21	1.99–2.20	3.42	2.90	172	1	2.95	
90°	–38.1	1.99–2.21	1.99–2.23	3.48	2.94	172			
180°	–48.3	1.99–2.21	1.99–2.19	3.45	2.96	172	2	2.87, 2.87	
270°	–39.8	1.99–2.21	1.99–2.23	3.47	2.95	171			
disp. 0°	–26.5	1.99–2.21	2.00–2.21	6.92		169			2.29
disp. 180°	–28.9	1.99–2.21	2.01–2.20	6.94		167			2.29
B97M-rV									
0°	–37.3	1.97–2.17	1.97–2.17	3.40	2.90	172	1	2.95	
90°	–35.1	1.97–2.17	1.97–2.19	3.43	2.90	171			
180°	–46.8	1.97–2.17	1.97–2.15	3.43	2.95	172	2	2.87, 2.87	
270°	–36.4	1.97–2.17	1.97–2.19	3.40	2.93	170			
disp. 0°	–17.9	1.97–2.17	1.98–2.17	6.81		165			2.24
disp. 180°	–19.6	1.97–2.17	1.99–2.16	6.87		167			2.24
B97-3c									
0°	–35.2	1.98–2.19	1.99–2.20	3.30	2.71	168	1	2.96	
90°	–31.8	1.98–2.19	1.98–2.19	3.37	3.03	172			
180°	–44.1	1.98–2.19	1.99–2.18	3.35	2.80	169	2	2.88, 2.88	
270°	–35.6	1.98–2.19	1.98–2.22	3.38	2.83	169			
disp. 0°	–19.7	1.98–2.19	1.98–2.19	6.81		169			2.45
disp. 180°	–33.4	1.98–2.19	1.99–2.19	3.42		169			
DFTB-D4									
0°	–33.6	1.99–2.27	2.01–2.24	3.17	2.56	167	1	2.87	
90°	–32.0	1.99–2.27	1.98–2.38	3.18	2.56	167			
180°	–39.6	1.99–2.27	2.02–2.22	3.21	2.63	168	2	2.82, 2.82	
270°	–32.4	1.99–2.27	2.01–2.24	3.17	2.52	163			
disp. 0°	–31.3	1.99–2.27	2.02–2.23	7.03		163			2.21
disp. 180°	–33.4	1.99–2.27	2.02–2.23	6.95		164			2.24
GFN2-xTB									
0°	–39.8	1.96–2.15	1.97–2.17	2.90	2.35	163	1	2.91	
90°	–38.4	1.96–2.15	1.97–2.21	2.90	2.37	163			
180°	–46.6	1.96–2.15	1.98–2.15	2.95	2.40	163	2	2.85, 2.85	
270°	–38.6	1.96–2.15	1.97–2.22	2.86	2.41	162			
disp. 0°	–40.3	1.96–2.15	1.96–2.16	6.99		167			2.11
disp. 180°	–40.1	1.96–2.15	1.97–2.14	6.93		170			2.24

The structure of the θ = 180° H-bonded dimer with two hydrogen bonds is similar to the structure crystalized by Barkigia et al.^47^ They observed that the dimers of Zn pheoporphyrin d orient to form nonlinear hydrogen bonds, similar to what we observed for molecule **3**. (155° for the dimer optimized at the B97M-rV/TZVP level of theory). In addition, Barkigia et al. found that Zn–O distances in the complexes are larger than 2.1 Å, which is also the case for the optimized dimer geometries in Table 5.

The displaced dimers have weaker π–π interaction and no hydrogen bonding, but they are stabilized by Zn–O metal–ligand interaction. As a result, most of the optimized geometries are close to the starting geometries except for the B97-3c optimization for the displaced geometry with θ = 180° rotation, where the monomers slip back into the close pack form where the Zn–Zn′ distance is 3.42 Å. Surprisingly, although the geometries for displaced dimers optimized with the DFTB-D4 and GFN2-xTB methods are similar to those obtained using the B97M-rV method, the BEs are very large, almost the same as the BE for the face-to-face dimers. This could be attributed to an overestimate of the metal–ligand interactions; in the geometries optimized with tight binding methods, the Zn–O and Zn–N′ bonds are shorter and N–Zn–N angles are smaller (doming).

Similar to the dimers of molecules **1** and **2**, the strongest interaction holding the molecule **3** dimers together is the dispersion, and consequently, the potential energy surfaces have wide minima. Therefore, any additional interaction such as metal–ligand or hydrogen bond and steric effects can modify the packing patterns, confirming the earlier findings of Mück-Lichtenfeld and Grimme for free-base porphyrin.^46^ These authors noted that the IE differences between different motifs stem from the electrostatics. The same is true for the dimers of molecule **3**. A previous work on the dimers of Chlorophyll *a* also had a similar conclusion.^10^

### IEs Depend on Substitutions

**Figure 5:**
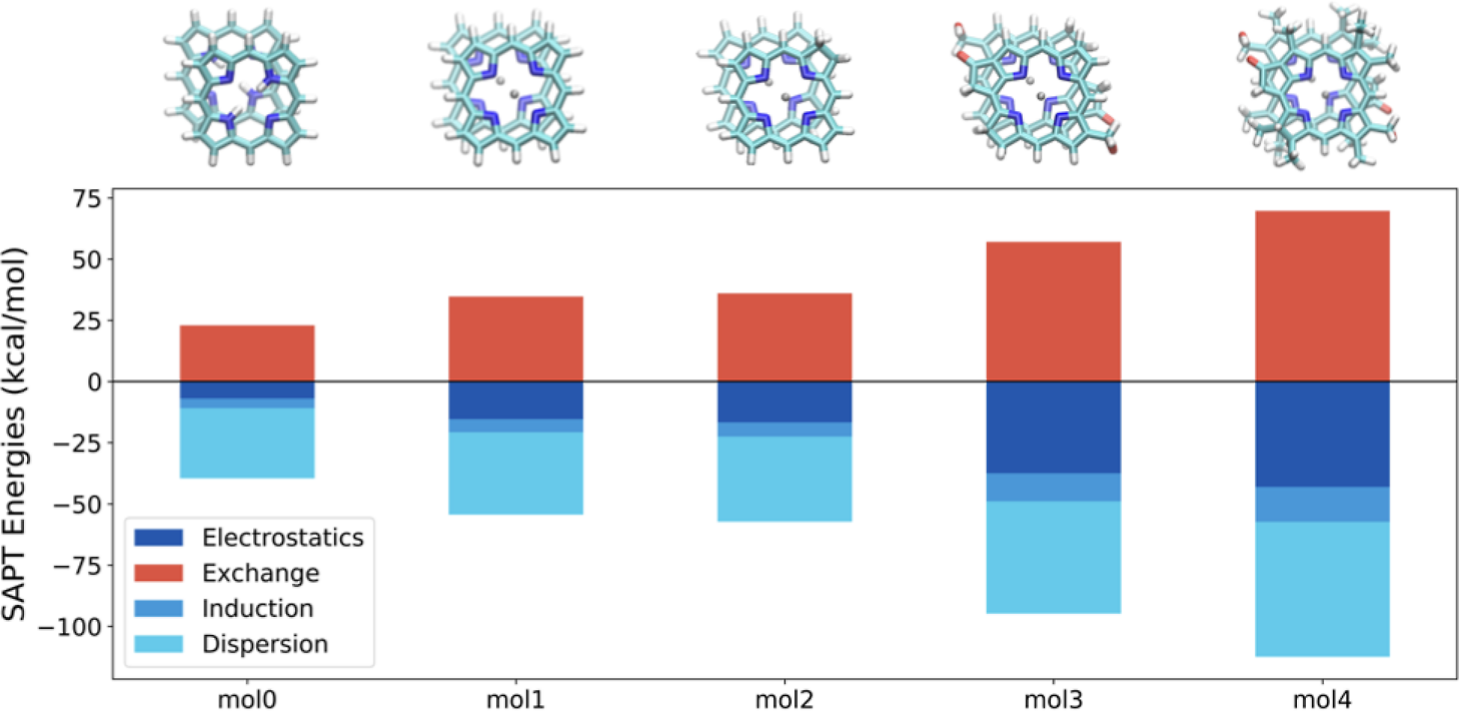
Decomposition of the IE with the SAPT(DFT)/def2-SVP level of theory for the dimers optimized at the B97M-rV/def2-TZVP level of theory.

To quantify the effect of substitutions on the IE, the dimers corresponding to the lowest BE conformers of molecules **1–3** were analyzed using the SAPT(DFT) method. In particular, we used the 90 and 180° rotated dimers for molecules **2** and **3**, respectively. In addition, we included two more dimers: (1) the free-base porphyrin dimer to investigate the effect of the Zn atom complexation (named molecule **0**. This is the most stable dimer configuration where the dimers are rotated 90°). (2) The 180° rotated dimer of molecule **4** to study the effect of the R-group substitutions. Similar to dimer 3, this dimer has two hydrogen bonding. The DFT and SAPT(DFT) IEs are compiled in Table 6. The decomposition of the IE into the components reveals that metal complexation and polar and R-group substitutions increase the IE (Figure 5).

**Table 6:** IEs for the Dimers Optimized at the B97M-rV/def2-TZVP Level of Theory (kcal/mol) (*c* Indicates BSSE Correction)

	B97M-rV	B97M-rV_*c*_	SAPT(DFT)	SAPT(DFT)
molecule	def2-TZVP	def2-TZVP	def2-SVP	def2-TZVP
0	–23.1	–21.6	–16.5	–23.9
1	–31.1	–29.2	–19.7	–29.7
2	–32.5	–30.2	–21.1	–30.1
3	–53.5	–50.0	–37.7	
4	–62.3	–58.6	–42.7	

**Figure 6:**
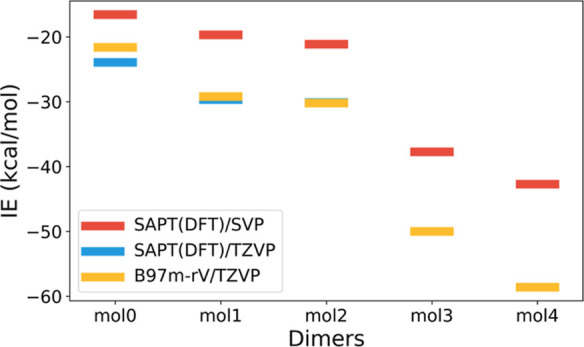
Comparison of IEs (kcal/mol) calculated with SAPT(DFT) and B97M-rV methods for the dimers optimized at the B97M-rV/def2-TZVP level of theory.

The BSSE-corrected IEs obtained using the B97M-rV/def2-TZVP level of theory are remarkably close to the SAPT(DFT)/def2-TZVP values for molecules **1** and **2**. On the other hand, although the def2-SVP basis underestimates the IE, the overall trends are more or less captured (Figure 6). A comparison of the SAPT results with these two bases reveals that the major error in def2-SVP stems from the inadequate description of the dispersion interaction (Table 7).

**Table 7:** Decomposition of the IE (kcal/mol) with the SAPT(DFT) Method for the Dimers Optimized at the B97M-rV/def2-TZVP Level of Theory

	SAPT/def2-SVP	elec.	exc.	ind.	disp.
0	–16.5	–6.9	22.9	–4.0	–28.6
1	–19.7	–15.3	34.7	–5.4	–33.7
2	–21.1	–16.6	36.1	–5.8	–34.7
3	–37.7	–37.4	57.0	–11.5	–45.9
4	–42.7	–43.0	69.7	–14.3	–55.2

	SAPT/def2-TZVP	elec.	exc.	ind.	disp.
0	–23.9	–7.2	24.0	–4.5	–36.2
1	–29.7	–17.9	39.6	–6.6	–44.8
2	–30.1	–16.6	37.3	–6.7	–44.2

For free-base porphyrin, Mück-Lichtenfeld and Grimme reported an IE value of −25 ± 3 kcal/mol. The values we obtained with def2-TZVP are similar, namely, −22 and −24 kcal/mol using DFT and SAPT methods, respectively.

Metal binding increases the dimer IEs by about −6 to −8 kcal/mol. We further analyzed the effect of metal binding in terms of the SAPT(DFT)/def2-TZVP components for molecules **1** and **2** (Table 7). Zn binding to the free-base porphyrin increases the electrostatics, dispersion, induction, and exchange interactions by −10.7, −8.5, −2.1, and 15.6 kcal/mol, respectively, resulting in an overall stabilization of dimers by approximately −6 kcal/mol. Furthermore, introducing a keto group and a hydroxy group further stabilizes the dimers by about −20 kcal/mol.

## Conclusions

Zinc binding in free-base porphyrin stabilizes the dimer significantly. Introducing the hydroxy and keto groups to the Zn-chlorin ring almost doubles the IE compared to that of the free-base porphyrin. Interaction surfaces for all parallel stacked dimers have mostly flat minima, where the rotation does not significantly change the energies. These flat interaction surfaces are perhaps the reason behind rich packing patterns observed in experimentally produced aggregates. All complexes are dispersion-bound, without which the BE would be positive. Although the dispersion is the strongest force, due to the flat PES, mostly electrostatics determines the geometries.

Most methods we investigated provide reasonably good geometries and capture the IE for the face-to-face dimers, consistently predicting the doubly hydrogen-bonded dimer as the lowest energy configuration. However, GFN2-xTB and DFTB-D4 predict the IE for displaced dimers to be as high as that for the face-to-face dimers, whereas in the DFT methods, the IE is smaller for the displaced dimers, as expected from the diminishing dispersion interactions. On the other hand, B97M-rV performs comparably with SAPT(DFT) at the same basis set level. In addition, the analysis showed that ignoring the geometry relaxation causes a more significant error than the BSSE.

Remarkable charge and energy transport properties emerge only for supramolecular aggregates. Therefore, understanding the molecular interactions at the aggregate scale for various packing patterns is underway in our laboratory.

## Supporting Information Available

Optimized geometries for the dimers (ZIP)
